# Remifentanil and Nitrous Oxide Anesthesia Produces a Unique Pattern of EEG Activity During Loss and Recovery of Response

**DOI:** 10.3389/fnhum.2018.00173

**Published:** 2018-05-07

**Authors:** Sarah L. Eagleman, Caitlin M. Drover, David R. Drover, Nicholas T. Ouellette, M. Bruce MacIver

**Affiliations:** ^1^Department of Anesthesiology, Perioperative and Pain Medicine, Stanford University, Palo Alto, CA, United States; ^2^University of Washington, Seattle, WA, United States; ^3^Department of Civil and Environmental Engineering, Stanford University, Stanford, CA, United States

**Keywords:** remifentanil, nitrous oxide, anesthesia, electroencephalography (EEG), consciousness

## Abstract

Nitrous oxide (N_2_O) and remifentanil (remi) are used along with other anesthetic and adjuvant agents for routine surgical anesthesia, yet the electroencephalogram (EEG) changes produced by this combination are poorly described. N_2_O administered alone produces EEG spectral characteristics that are distinct from most hypnotics. Furthermore, EEG frequency-derived trends before and after clinically relevant time points vary depending on N_2_O concentration. Remifentanil typically increases low frequency and decreases high frequency activity in the EEG, but how it influences N_2_O’s EEG effect is not known. Previous attempts to characterize EEG signals of patients anesthetized with N_2_O using frequency-derived measures have shown conflicts and inconsistencies. Thus, in addition to determining the spectral characteristics of this unique combination, we also test whether a newly proposed characterization of time-delayed embeddings of the EEG signal tracks loss and recovery of consciousness significantly at clinically relevant time points. We retrospectively investigated the effects of remi and N_2_O on EEG signals recorded from 32 surgical patients receiving anesthesia for elective abdominal surgeries. Remifentanil and N_2_O (66%) were co-administered during the procedures. Patients were tested for loss and recovery of response (ROR) to verbal stimuli after induction and upon cessation of anesthesia, respectively. We found that the addition of remifentanil to N_2_O anesthesia improves the ability of traditional frequency-derived measures, including the Bispectral Index (BIS), to discriminate between loss and ROR. Finally, we found that a novel analysis of EEG using nonlinear dynamics showed more significant differences between states than most spectral measures.

## Introduction

Nitrous oxide (N_2_O), an NMDA antagonist, has been used since the mid 1800s, and is commonly used today both for painful diagnostic and dental procedures (Rampil et al., 1998) and as a supplement to other anesthetic medications such as propofol and halogenated ethers like sevoflurane (Pavone et al., 2016). Several studies have characterized the influences of N_2_O on EEG signals in both patients and volunteers. Generally, N_2_O shows reductions in total power predominately at frontal sites in humans during induction and an increase in total power upon recovery (Foster and Liley, 2011, 2013). The increase in total power during induction has been attributed to increases in power in the delta and theta bands (Foster and Liley, 2011). N_2_O has also been reported to maintain awake-like power in the alpha band after induction (Foster and Liley, 2011). Furthermore, an increase in theta power during washout/withdrawal prior to recovery has also been reported (Rampil et al., 1998; Foster and Liley, 2011). Conflicting evidence exists as to whether N_2_O increases high frequency (beta and gamma) activity (Yamamura et al., 1981; Rampil et al., 1998) following induction or simply maintains it (Foster and Liley, 2011, 2013). However, this conflicting evidence may be related to dose-dependent changes as those that reported an increase in beta and gamma used much higher concentrations of N_2_O (Yamamura et al., 1981; Rampil et al., 1998; 50%–70%) than those who reported that power in these frequencies was maintained (Foster and Liley, 2011, 2013; 20%–40%). Adding N_2_O to propofol anesthesia has been shown to interfere with BIS measures by increasing BIS values (indicating awakening) with increasing N_2_O levels and depths of anesthesia (Doufas et al., 2003).

Remifentanil (remi), a potent, short-acting opioid, has a faster clearance after discontinuance than other opioids such as alfentanil (Egan et al., 1996). Like other opioids, remi is most commonly used with other agents. Remi has been shown in frontal and parietal regions of rats to slow the EEG activity with increases in delta activity and intermittent bursts of increased theta activity following anesthetic induction (Lozito et al., 1994). When combined with propofol for sedation, remi decreases the amount of anesthetic needed and can allow for lighter sedation levels while maintaining patient comfort (Mazanikov et al., 2011). In humans the same slowing of EEG activity from high frequency, low amplitude signals to low frequency, high amplitude signals can be observed (Egan et al., 1996). Furthermore, increases in total power and delta power and decreases in beta power have been described when remi is used together with propofol (Kortelainen et al., 2009). Spectral edge frequency (SEF95), the frequency that bounds 95% of the power from above, is a useful measure to determine the depth of anesthesia. SEF95 decreases with administration of remi alone in human volunteers (Egan et al., 1996). When remi was combined with propofol during induction of anesthesia for surgical procedures, a similar decrease in SEF95 was observed after patients lost response to verbal commands (loss of response, LOR); however, addition of remi (compared to propofol only) attenuated this effect in a dose-dependent manner (Kortelainen et al., 2008). Addition of remi to propofol anesthesia also appears to attenuate the increase in delta activity following LOR compared to the administration of propofol alone (Kortelainen et al., 2009). Overall, there are mixed reports that remi modulates the EEG effects produced by propofol (Koitabashi et al., 2002; Mustola et al., 2005; Ferenets et al., 2007; Kortelainen et al., 2008) and other agents like sevoflurane (Olofsen et al., 2002; Manyam et al., 2007). More importantly, though both N_2_O and remi are used separately and in combination with other anesthetic and adjuvant agents for routine surgical anesthesia, little is known about their combined effects on EEG activity.

The use of EEG monitoring in anesthetic clinical practice has been limited by the nuances and inconsistencies in both patients and anesthetic agents. Neither patients nor different agents display repeatable responses, and this can produce conflicting results in frequency analyses. Inconsistent results call for more detailed studies and for applying the same spectral analysis techniques to all possible combinations and titrations of anesthetic and adjuvant agents. However, in order to make EEGs more practical and universal for anesthesiologists, these results also call for the development of new analytical techniques that are anesthetic and patient invariant. One possible analysis opportunity is the application of techniques developed in the nonlinear dynamics community to extract dynamical attractors during different anesthetic states. Such attractors can be created with as little as a single recording electrode and can be computed in seconds. The utility of this kind of analysis has been explored previously with EEGs collected from patients undergoing anesthesia (Watt and Hameroff, 1988; Walling and Hicks, 2006) and in microEEGs from anesthetized rats (MacIver and Bland, 2014). All these studies observed changes in attractor shape following anesthetic induction and during recovery. Two-dimensional and three-dimensional attractors (where the dimensionality depends on the embedding protocol) change from circular/spherical shapes to more elliptical/ellipsoidal ones. Walling and Hicks (2006) proposed a way to quantify the changes exhibited in attractors upon recovery from anesthesia (2006); however, the method they introduced had a high error rate for recovery of consciousness. Additionally, no one has yet compared the same measurement across all anesthetic states, nor explored the results with other commonly used spectral analyses. Whether similar 3D attractor shape changes can be observed with different anesthetics and adjuvant agent combinations needs to be explored.

To better understand the spectral changes that occur with co-administration of N_2_O and remi we conducted several standard frequency domain measures: spectral edge frequency, total power, and percentage of power in different frequency bands. We also compared these measures to the Bispectral Index (BIS) values to determine how the addition of remi to N_2_O anesthetic protocol affected the BIS value. BIS is a spectral measure generated by the BIS brain monitor technology used to monitor anesthetic depth, but does not perform well during N_2_O anesthesia (Rampil et al., 1998; Barr et al., 1999; Hirota et al., 1999; Doufas et al., 2003). Finally, we tested whether similar shape changes could be observed during anesthetic administration in time-delayed embeddings with this unique anesthetic combination. Additionally, we compared spectral and BIS values with our new characterization measure of time-delayed embeddings. We performed measurements around clinically relevant time points during anesthetic exposure: before and after LOR to verbal stimuli (consciousness), during the deepest level of anesthesia, and before and after recovery of response (ROR) to verbal stimuli.

## Materials and Methods

### Study Protocol

This study was carried out in accordance with the recommendations of Stanford School of Medicine Administrative Panel on Human Subjects in Medical Research. The protocol was approved by the Panel on Human Subjects in Medical Research. All subjects gave written informed consent in accordance with the Declaration of Helsinki. The database consisted of 40 surgical all comers (20 males and 20 females) who were undergoing anesthesia for elective intraabdominal procedures classified as status 1–3 by the American Society of Anesthesiologists. We retrospectively reviewed the database and analyzed data under a separate Stanford Approved IRB. Patients were not pregnant or breast feeding and were within 35% of ideal body weight. Patients with history of substance abuse, seizures, severe respiratory disease, renal or hepatic dysfunction, congestive heart failure or symptomatic ischemic heart disease were excluded from the study (Drover and Lemmens, 1998). Also, patients were not exposed to opioids within 9 h and not exposed to barbituates within 24 h of the start of the study (Drover and Lemmens, 1998).

A detailed description of surgical procedures and anesthetic administration have been previously described (Drover and Lemmens, 1998). Briefly, patients received 1-to 2-mg intravenous dose of Midazolam an average of 26 (±14) min (minimum 6 min, maximum 80 min) prior to the onset of anesthesia. After the midazolam administration, a 20-gauge radial artery catheter was inserted to collect blood samples to measure remi blood concentrations during the procedure. After the procedure, blood concentrations were determined using liquid chromatography (Drover and Lemmens, 1998). Pancuronium was administered 0.02-mg/kg intravenous dose 3 min or more prior to induction. Patients were anesthetized with varying levels of remifentanil with a steady background of 66% N_2_O. Remifentanil was administered with an infusion pump controlled by STANPUMP software^1^ running on a laptop computer. Patients were tested for verbal response to verbal stimuli after beginning N_2_O and infusion of remi and the physician noted when patients lost response to verbal stimuli (LOR). After LOR, succinylcholine was administered 1 mg/kg intravenous dose prior to intubation. After the surgical procedure was completed and N_2_O and remi were discontinued, ROR was determined when patients made purposeful response to verbal stimuli.

Out of the original 40 patients, we used 32 for our analysis (16 males and 16 females). Eight patients were excluded because we could not extract consistent artifact-free clips in their electroencephalograms (EEGs) around our time points of interest (discussed below). Patients ranged in age from 28 years old to 75 years old (mean age of 53 years old with a standard deviation of 13).

### EEG Recording and Preprocessing

**Figure 1:**
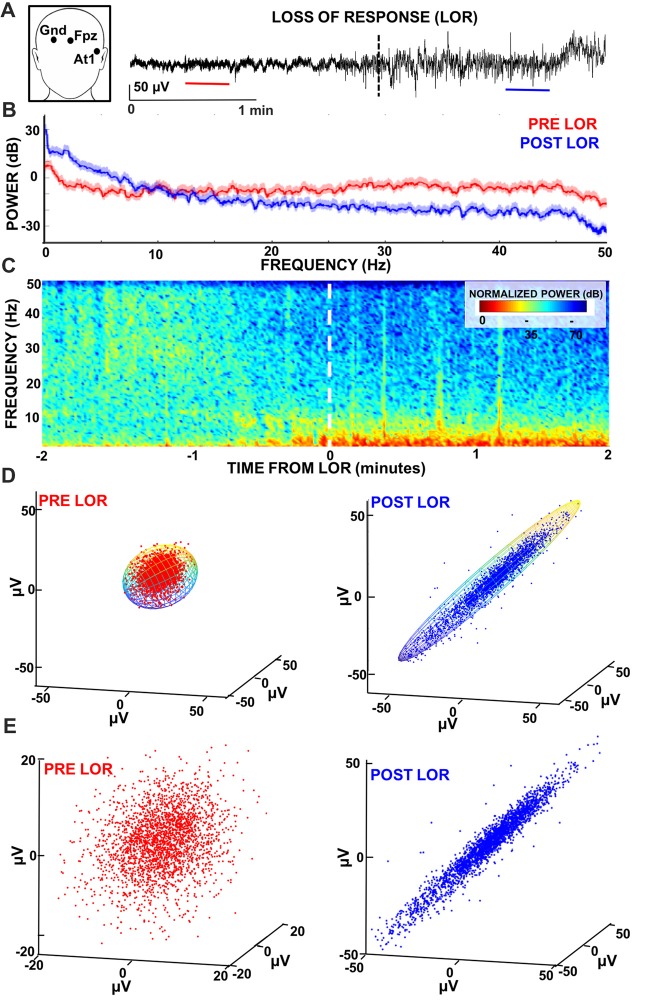
Characterizing loss of response (LOR) using electroencephalogram (EEG) analyses. **(A)** EEG recordings were made from At1 referenced to Fpz. We examined 2 min windows before and after LOR to verbal stimuli (indicated by dashed line) to identify 20 s artifact free clips (pre LOR clip shown in red, post LOR clip shown in blue) for subsequent analysis. **(B)** Multitaper spectral results of the 20 s clips before (red) and after (blue) LOR. An increase in delta and theta activity as well as a reduction in beta and gamma frequencies is observed post LOR compared to the pre LOR. **(C)** Normalized spectrograms (highest power is 0 dB) of the EEG activity during a 3 min window surrounding the LOR event. An increase in low frequency delta and theta activity and a decrease in higher frequency amplitudes (beta and gamma) is observed starting approximately half a minute prior to LOR. **(D)** Time-delayed embeddings were plotted from pre (red) and post (blue) LOR 20 s clips using embedding delays of 5 ms. Embeddings were fit with 3D ellipsoids (note: ellipsoid cage is multicolored to show the viewing angle of the attractors is the same). **(E)** Same as **(D)** but attractors are autoscaled to the maximum dimension per condition to show the differences in the radius ratios that we measured. We use this autoscaling in attractors plotted from the same viewing angle in subsequent figures to best illustrate the shape changes we observe. Data presented in this figure is from Case 24.

An A-1000 EEG monitor (Aspect Medical Systems, Natick, MA, USA) was attached to the patients using adhesive electrodes prior to starting the anesthetic as per the manufacturer’s instructions. EEGs were recorded at At1 (a recording position more lateral and ventral to F7) referenced to Fpz (Figure 1A) at 128 Hz. The referential montage of At1-FpZ was recommended by the manufacturer of the A-1000 to reduce the electrocardiogram artifacts during periods of suppression and make the BIS numbers more reliable. Records of the case events including time stamps of start of induction, LOR to verbal stimuli, anesthetic concentrations, anesthetic discontinuation, and ROR to verbal stimuli were de-identified and then used for analysis. EEGs were detrended and notch filtered using a 2nd order Butterworth IIR filter to remove 60 Hz noise prior to analysis.

Twenty second artifact-free clips occurring within 2 min before and after LOR and within 2 min before and 4 min after ROR were manually selected for subsequent analysis (Figure 1A). A larger time window was inspected for ROR clips because there were no continuous 20 s clips post recovery of consciousness within 2 min for some cases. Extending the selection of clips in the post recovery period to 4 min allowed us to analyze a comparable number of sessions (*n* = 29) for both time periods of interest (22 patients were the same for both LOR and ROR groups). Three of the authors (CMD, SLE, DRD) visually inspected all EEG data for each patient to ensure clips selected for subsequent analysis were free of noise and artifacts.

Since we had blood concentrations of remifentanil during the procedure, we took 20 s clips within 2 min prior to the measured sample of the highest concentration of remifentanil in each case to represent a point at which patients were most anesthetized (High-Remi). The average measured remi concentration was 12.7 ± 6.2 ng /ml for these clips (an outlier blood concentration for 1 patient was removed). For this condition, we used cases that had either LOR and ROR clips (*n* = 32) as we wanted to test if our measures were sensitive to the difference between anesthetic states once the patients were already anesthetized. We made comparisons for these deeply anesthetized periods and LOR and ROR events in the same cases.

### Spectral Analyses

**Figure 2:**
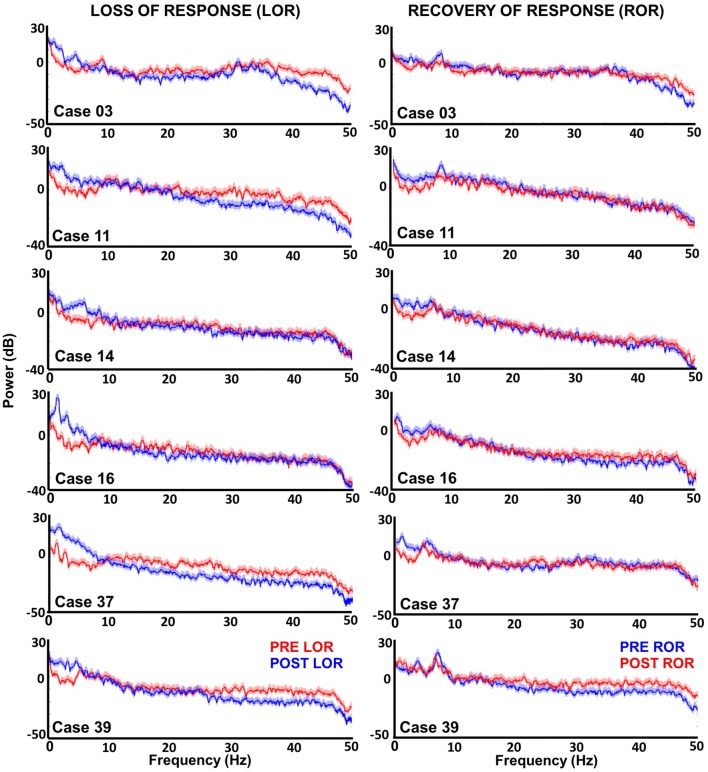
Multitaper spectral analysis of six patients before and after loss and recovery of verbal responses. Periods where patients do not respond to verbal stimuli are shown in blue; whereas, periods where responses are present are in red. For most patients increases lower frequencies (delta and theta) are observed along with decreases in higher frequencies (beta and gamma) after LOR. However, Cases 14 and 16 show that not all patients exhibit the reduction in higher frequencies. The differences between intact and not intact responses are less obvious in the recovery of response (ROR) spectra. During ROR we observe decreases in lower frequencies (delta and theta) for most patients; however, the increase in higher frequencies post ROR is less obvious as spectra appear to overlap except in the example from Case 39. We also observed a peak in theta (~9 Hz) in the pre ROR clips.

We performed multitaper spectral analysis on the 20 s manually selected artifact-free clips during pre and post LOR and pre and post ROR using the MATLAB Chronux toolbox (Mitra and Bokil, 2008; example spectra Figures 1B, 2)^2^. Specifically, we used a time-bandwidth product of five with nine tapers, limited the frequency ranges calculated to 0–50 Hz, and computed the theoretical error range at the 95% confidence interval. Power values were converted from magnitude to decibels.

**Figure 3:**
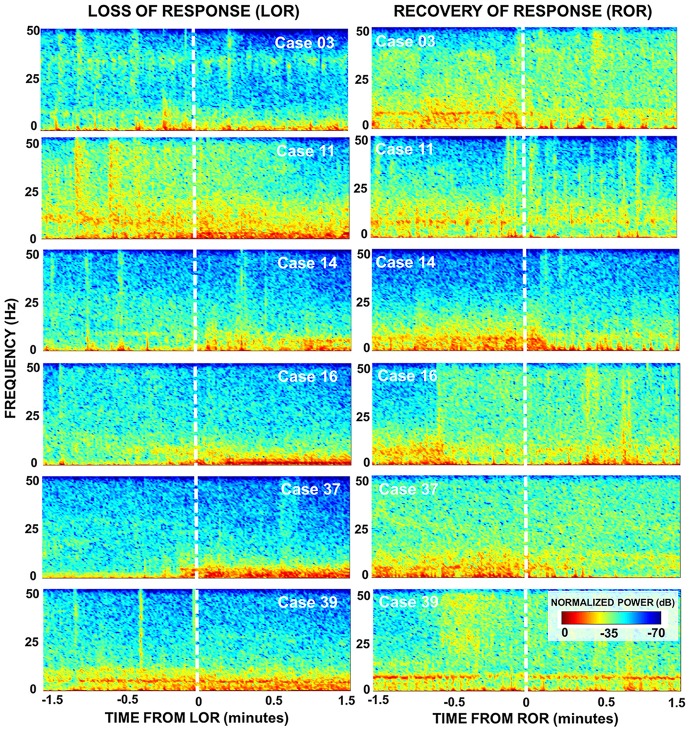
Spectral dynamics during LOR and ROR to verbal stimuli. Normalized spectrograms depict the dynamics in brain activity of six patients during 3 min around LOR (left) and ROR (right). An increase in lower frequencies (delta and theta) post LOR is observed for most patients as well as a subtle decrease in higher frequencies (beta and gamma). The power in the lower frequency bands is reduced after ROR as well as increased power in the higher frequency bands. During the pre ROR period we also see the presence of a theta rhythm (~9 Hz) that disappears around the time of ROR in most patients. For both transition periods the temporal dynamics of these changes vary.

To visualize the temporal profile of the spectral changes that occurred during the windows surrounding the LOR and ROR transitions, we computed normalized spectrograms (Figures 1C, 3). We created a custom script that calculates the Fourier transform in Hann windows with half window overlaps. We then cut off the frequencies above 50 Hz, converted the magnitude to decibels (dB), and scaled the spectrogram output by the maximum magnitude, so that the maximum power was 0 dB.

We calculated the spectral edge frequency (i.e., the frequency bounding 95% of the power from above, SEF95) and total power from our multitaper spectral analysis. Total power results were converted to decibels. To make our results comparable to previous work, we calculated SEF95 and total power both for the full range of frequencies available given our acquisition system sampling rate and with a band-limited range between 0 Hz and 30 Hz. In addition, we calculated the percentage of total power for individual frequency bands per condition. The percentage of total power was used because previous research (Drummond and Patel, 2005; Foster and Liley, 2011) has reported significant changes in total power with exposure to anesthetics. The ranges we used for the frequency bands were as follows: delta: 0.1–4 Hz; theta: 4–9 Hz; alpha: 9–12 Hz; beta: 12–30 Hz; and gamma: 30–60 Hz (Kortelainen et al., 2008, 2009; Foster and Liley, 2011, 2013).

### BIS and EMGlow Values

Since EEG data was collected on an A-1000 EEG monitor (Aspect Medical Systems, Natick, MA, USA), we were able to obtain the BIS and EMGlow values corresponding to the same 20 s clips we used for our analysis. BIS values represent an index provided by the manufacturer that is intended to guide clinicians during anesthesia titration. The EMGlow value is another value generated by the manufacturer that gives the absolute power in the 70–110 Hz range. The manufacturer implies that this represents EMG activity; however, as it is used here (recorded from the same scalp electrode) it could also be interpreted as high gamma (Rampil et al., 1998; Ortolani et al., 2002; Muthukumaraswamy, 2013). Note that our EEG sampling frequency of 128 Hz limited us from comparing this value to one we could calculate from the EEG signal ourselves. The values we report are averages over the 20 s clips. Note that this data was recorded on a version of the A-1000 monitor from 1995 and may not accurately reflect the BIS or EMGlow numbers generated by newer versions of the EEG monitor.

### Characterization of Dynamical Attractors

We constructed three-dimensional time-delayed embeddings (attractors) of the EEG signal as previously described (Watt and Hameroff, 1988; Walling and Hicks, 2006; MacIver and Bland, 2014) using a 5 ms delay (Figures 1D,E, 6). Previous work has illustrated shape changes in the 3D attractors after anesthetic administration and following recovery (Watt and Hameroff, 1988; Walling and Hicks, 2006; MacIver and Bland, 2014). Specifically, more spherical attractors are seen prior to anesthetic exposure that appear to become ellipsoidal when subjects are anesthetized (Watt and Hameroff, 1988; Walling and Hicks, 2006; MacIver and Bland, 2014). This three-dimensional shape change has only been observed in a handful of studies, but not compared across all anesthetic states. This work is an attempt to quantify this effect to determine if it meaningfully characterizes anesthetic state compared to other clinical EEG measures. We quantified this shape change by fitting the three-dimensional attractor to an ellipsoidal solid of revolution (Khachiyan, 1980; Figure 1D). The lengths of the symmetry axes of the ellipsoid were calculated and the ratio of the minimum and maximum axes (which we term the radius ratio) was used to quantify the shape change. A radius ratio of 1 implies a sphere, while smaller ratios imply more strongly ellipsoidal shapes.

**Figure 4:**
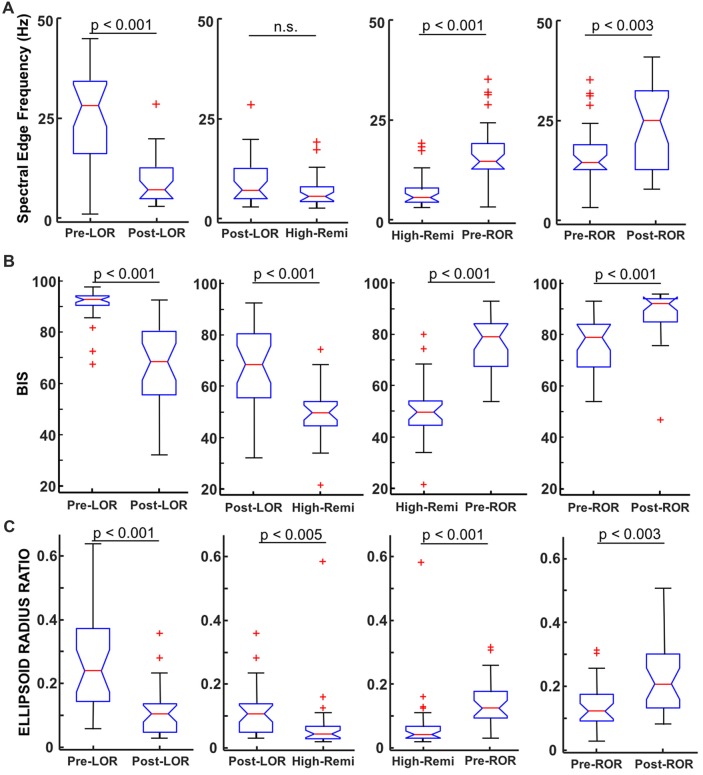
Comparison of spectral edge frequency, Bispectral Index (BIS) values and ellipsoid radius ratio to distinguish anesthetic depth. **(A)** Spectral Edge Frequency (SEF95), the frequency at which 95% of the power is below is shown for pre- and post-LOR, High Remi and pre- and post-ROR. SEF95 showed significantly different changes pre- and post- LOR and ROR and between High-Remi and pre-ROR. SEF95 did not show a significant difference between post-LOR and High-Remi. **(B)** BIS showed significant differences between all conditions. **(C)** Our novel analysis, ellipsoid radius ratio, showed significant differences for all of our comparisons as well. Box and whisker plots show the median (red line), 25th and 75th quartiles (bottom and top edges of the blue box, respectively), most extreme data points (error bars), and outliers (red plus signs).

**Figure 5:**
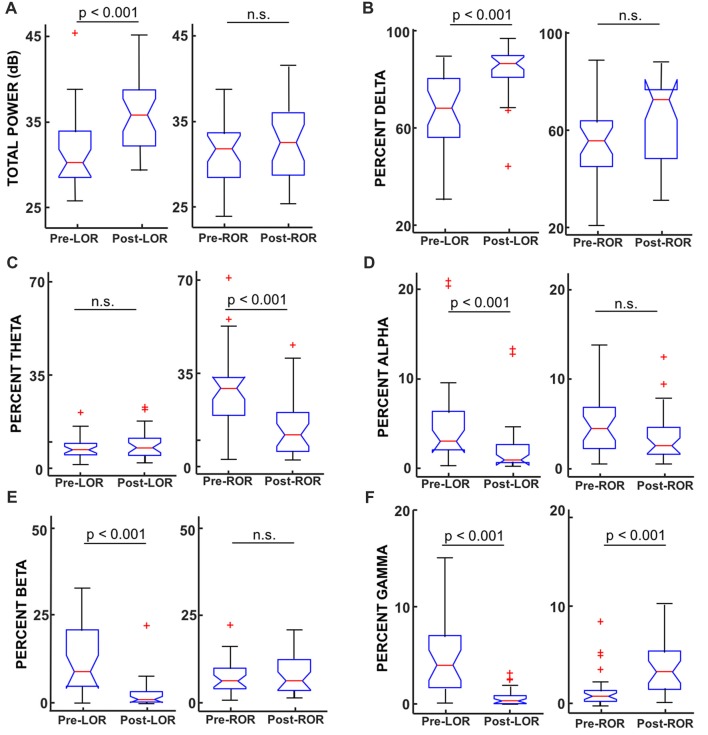
Total power and percentage of total power in band-limited ranges before and after LOR and ROR. **(A)** Total power (from full spectrum, in dB) showed a significant increase from pre- to post-LOR but did not show any significant difference pre- to post-ROR (*p* = 0.21). **(B)** Percent delta power (0.1–4 Hz) showed a significant increase pre- to post-LOR but did not show a significant different pre- and post-ROR (*p* = 0.04). **(C)** Percent theta power (4–8 Hz) did not show a significant change pre- and post-LOR (*p* = 0.66) but did show a peak at pre-ROR (greater there than any other time point). The percent theta pre-compared to post-ROR was significantly less. **(D)** Percent alpha power (9–12 Hz) decreased significant pre- and post-LOR, and showed a non-significant trending decrease between pre- and post-ROR (*p* = 0.05). **(E)** Percent beta power (12–30 Hz) decreased between pre- and post-LOR but did not show a significant change pre- and post-ROR (*p* = 0.34). **(F)** Percent gamma power (30–60 Hz) showed a significant decrease pre- to post-LOR and a significant increase pre- to post-ROR. Box and whisker plots show the median (red line), 25th and 75th quartiles (bottom and top edges of the blue box respectively), most extreme data points (error bars), and outliers (red plus signs).

**Figure 6:**
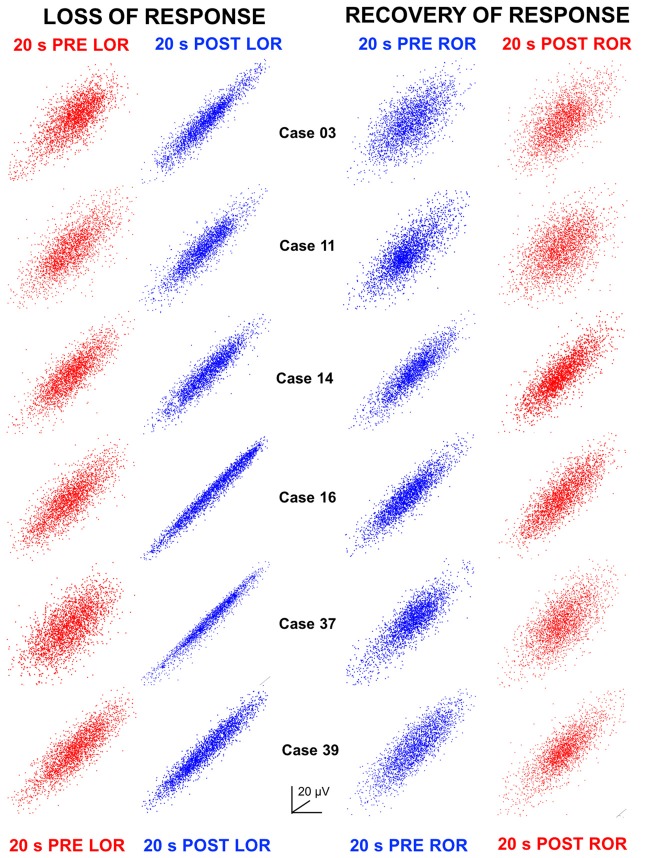
Attractors at LOR and ROR. Three-dimensional time-delayed embeddings (attractors) of the 20 s EEG clips at 5 ms delays are plotted here as two-dimensional point clouds. Attractors have been oriented to the same azimuth and elevation per case but have been auto-scaled per attractor in the x, y and z axes to the maximum amplitude (see Figures 1D,E for scaling and dimensionality to the sizes when compared at the same vs. auto-scaled axes). Attractors during awake, responsive states are shown in red; whereas, attractors during anesthetized, unresponsive states are shown in blue. Here we show clearly that we observe the same flattening in the attractors in all patients after LOR to verbal stimuli. Some flattening was already occurring in the pre LOR period as patients take an average of 4 (±1.4) min to lose consciousness after induction and thus some anesthetic effect was starting to be observed here. The thicker more spheroidal shape is recovered during ROR and can be seen when comparing the pre- vs. post-ROR attractors. We also observe that attractors are less flat at pre ROR compared to post LOR. This shows that our analysis captures the differences observed previously that the dynamics when anesthesia is induced are very different than those observed during recovery from anesthesia.

### Statistical Analysis

We performed a Kruskal-Wallis test with *post hoc* Wilcoxon Signed Rank tests to determine if there were significant differences between the following comparisons: pre- and post-LOR, post-LOR and High-Remi, High-Remi and pre-ROR, and pre- and post-ROR. We chose nonparametric tests because our data were not normally distributed (determined using Lilliefor’s test). Given the well-established differences in LOR and ROR brain states (neural inertia; Friedman et al., 2010), we considered these periods to be independent and incorporated the Bonferroni correction method for multiple comparisons because we were comparing these time periods to the period where remifentanil was the highest (High-Remi). Thus, we considered *p*-values below 0.0125 to be significant.

## Results

### Spectral Changes

For individual examples, we chose six representative patients that had artifact-free clips both before and after LOR and before and after ROR. In the spectra of most patients, we saw an increase in lower frequency activity following anesthetic administration and a decrease in higher frequency activity (Figure 2), consistent with effects seen using other hypnotic/anesthetics. However, there was no decrease in higher frequency activity for patients 14 and 16; the spectra for only these patient examples overlap in the higher frequency range before and after LOR (Figure 2). During the ROR period we observed the opposite trend, where a reduction in power of lower frequencies is observed after patients recover their response to verbal stimuli, accompanied by an increase of power of high frequencies (Figure 2). Here again we observed some variability in responses to anesthetics: cases 3 and 39 do not show a similar reduction in delta activity upon emergence from anesthesia. Interestingly, cases 3 and 39 appear to show the greatest increase in higher frequencies (Figure 2).

To look at the time-course of the spectral changes surrounding LOR and ROR, we computed the spectrograms of the EEG activity within a 3-min window surrounding the loss and ROR (Figure 3). Here we also saw increases in low frequency activity as well a decrease in high frequency activity in most patients. As in the spectra we also observe idiosyncrasies between patients in their responses. The noticeable changes in the spectral characteristics happen at varying times relative to the behavioral LOR. Cases 16 and 37 have abrupt changes in low frequency activity following LOR; however, Cases 39 and 11 appear to have gradual changes already occurring before they lose their response to verbal stimuli (Figure 3).

To quantify these observations, we first computed the spectral edge frequency (SEF95) for each of our time points, including the period of highest remifentanil concentration that we considered to be our most deeply anesthetized state (High-Remi). We show our results for our full spectrum in Figure 4A and report results for the band-limited range. Reported results are medians [25, 75 percentiles] and significance values (*p*) are from Wilcoxon Signed Rank Tests with Bonferroni correction. We found that full spectrum SEF95 showed highly significant differences between pre- and post- LOR (*p* < 0.0001). Lesser but still significant differences between pre- and post-ROR (*p* = 0.0026, Figure 4A) were observed. No significant difference was found between High-Remi and post-LOR (*p* = 0.10), but a significant difference between High-Remi and pre-ROR (*p* < 0.0001, Figure 4A) was observed. For the band-limited (between 0 Hz and 30 Hz) SEF95 we observed a significant decrease between pre- and post-LOR (pre-LOR 19.5 [12.2, 24.6], post-LOR 5.9 [4.7, 9.6], *p* < 0.0001). In this range, we did not observe a significant difference between post-LOR and High-Remi (High-Remi_LOR_ 5.5 [4.2, 7.8], *p* = 0.23). We observed a significant increase in the High-Remi to pre-ROR (High-Remi_ROR_ 5.4 [4.2, 7.8], pre-ROR 13.3 [11.1, 16.0], *p* < 0.0001), but not a significant change between pre- and post-ROR (post-ROR 15.2 [9.6, 19.6], *p* = 0.11).

To investigate the specific frequency bands that underlie these differences we calculated and compared the total power between these anesthetic conditions. We show results for LOR and ROR before and after transitions (Figure 5) and report results for post-LOR and High-Remi and High-Remi and pre-ROR comparisons. Reported results are median [25, 75 percentiles]. Significance values (*p*) are again from post Wilcoxon Signed Rank Tests with Bonferroni corrections. We found a significant difference between pre- and post-LOR full spectrum total power (*p* < 0.0001, Figure 5A) but not a significant difference between pre-and post-ROR (*p* = 0.21, Figure 5A). We found that post-LOR was not significantly different from High-Remi (post-LOR 3.8 [3.0, 7.3] dB, High-Remi_LOR_ 3.7 [3.0, 5.7] dB, *p* = 0.45), but High-Remi and pre-ROR were significantly different (High-Remi_ROR_ 3.5 [3.0, 5.7] dB, pre-ROR 11.0 [8.1, 13.3] dB, *p* < 0.0001). In the band-limited (0–30 Hz) total power calculation we observed a significant decrease in total power between pre- and post-LOR (pre-LOR 30.0 [28.3, 33.8], post-LOR 35.7 [32.1, 38.5], *p* < 0.0001), but no significant difference between pre- and post-ROR (pre-ROR 31.7 [28.1, 33.6], post-ROR 32.4 [28.5, 36.0], *p* = 0.24). We did not observe a significant difference between post-LOR and High-Remi (High-Remi_LOR_ = 35.3 [32.6, 36.7], *p* = 0.26), but did observe a significant difference between High-Remi and post-ROR (High-Remi_ROR_ 35.2 [32.6, 36.4], *p* < 0.0003). We then calculated the percentage of total power in band-limited ranges. We found significant increases in delta activity (0.1–4 Hz) between pre- to post-LOR (*p* < 0.0001, Figure 5B) and a trend (not significant, *p* = 0.037) of increasing delta pre- to post-ROR (Figure 5B). There was no significant difference between the percentage of delta activity between post-LOR and High-Remi (post-LOR 86.8 [81.0, 90.0], High-Remi_LOR_ 89.5 [82.2, 92.7], *p* = 0.44), but a significant increase between High-Remi and pre-ROR during recovery from anesthesia (High-Remi_ROR_ 89.5 [82.2, 92.7], pre-ROR 55.6 [45.1, 63.8], *p* < 0.0001). In the theta band (4–9 Hz) we observed no significant changes in the activity except for a significant increase in theta in the pre-ROR clip (pre- and post-LOR *p* = 0.66, post-LOR 6.9 [4.1, 10.5], High-Remi_LOR_ 6.6 [4.0, 11.8], *p* = 0.41, Figure 5C). The pre-ROR period was significantly higher than High-Remi and post-ROR (High-Remi_ROR_ 6.3 [4.0, 11.8], pre-ROC 28.6 [18.5, 32.6], *p* < 0.0001, post-ROC 15.0 ± 11.7, *p* = 0.0003 [compared to pre-ROC]). We observed a significant decrease in the alpha band (9–12 Hz) between pre- and post-LOR (*p* = 0.0004, Figure 5D) and no significant difference between pre- and post-ROR (*p* = 0.05, Figure 5D). There was no significant change between post-LOR and High-Remi (post-LOR 0.8 [0.5, 2.5], High-Remi_LOR_ 0.6 [0.4, 2.0], *p* = 0.41), but a significant increase in alpha between High-Remi and pre-ROR (High-Remi_ROR_ 0.6 [0.4, 2.0], pre-ROR 4.5 [2.2, 6.9], *p* < 0.0001). Beta (15–30 Hz) and gamma (30–60 Hz) were the frequency bands in which we saw significant changes during the pre- and post-LOR period (Figures 5E,F). In the beta band we observed a significant decrease between pre- to post-LOR (*p* < 0.0001, Figure 5E) but no significant change between pre- and post-ROR (*p* = 0.34, Figure 5E). We observed no change between post-LOR and High-Remi (post-LOR 1.2 [0.5, 3.5], High-Remi_LOR_ 1.1 [0.5, 1.8], *p* = 0.29), but did see a significant increase in percentage of beta between pre-ROR and High-Remi (pre-ROR 6.4 [4.2, 10.0], High-Remi_ROR_ 1.1 [0.6, 1.8], *p* < 0.0001). In the gamma band we observed a decrease in the percentage of gamma from pre- to post-LOR (*p* < 0.0001, Figure 5F) and a significant increase in gamma from pre- to post-ROR (*p* < 0.0001, Figure 5F). We also observed a significant decrease in gamma between post-LOR and High-Remi (post-LOR 0.4 [0.1, 0.9], High-Remi_LOR_ 0.1 [0.08, 0.2], *p* = 0.0027; High-Remi_ROR_ 0.1 [0.08, 0.2], pre-ROR 1.1 [0.5, 1.7], *p* = 0.0001).

### BIS and EMGlow Changes

Since EEG data were recorded on an A-1000 EEG monitor we were able to obtain the BIS numbers corresponding to the same 20 s clips that we were analyzing; BIS numbers were averaged over the 20 s. We made the following comparisons to see how well BIS was able to identify differences in anesthetic depth: pre- and post- LOR, post-LOR and High-Remi, High-Remi and pre-ROR, and pre- and post-ROR (Figure 4B). We found that BIS numbers were significantly different in these comparisons (Figure 4B) based on Wilcoxon Signed Rank Tests with Bonferroni correction. BIS numbers decreased from pre- to post- LOR (*p* < 0.0001) and again significantly decreased from post-LOR to High-Remi (*p* < 0.0001). We observed increased BIS values from High-Remi to pre-ROR (*p* < 0.0001) and again significant increases in BIS values pre- to post-ROR upon recovery (*p* = 0.0006). This trend in BIS numbers is what is expected from the manufacturer’s manual during a typical timeline for anesthesia.

In addition to the BIS value, the A-1000 EEG monitor also outputs a value that it labels EMGlow which is said to be the absolute power in the 70–110 Hz range and related to EMG activity. EMGlow is calculated from the same EEG scalp electrodes used to record the EEG activity. We extracted these numbers as well from the same time as the 20 s clips and averaged the values. We found EMGlow numbers were significantly different in these comparisons. Values are reported as median [25, 75 percentiles] and significance was assessed using Wilcoxon Signed Rank Test with Bonferroni correction. EMGlow values significantly decreased from pre- to post-LOR (pre-LOR 44.1 [38.9, 49.3], post-LOR 36.9 [30.2, 40.0], *p* < 0.0001), and again from post-LOR to High-Remi (High-Remi_LOR_ 27.8 [27.0, 31.6], *p* < 0.0006). EMGlow values then increased from High-Remi to pre-ROR (High-Remi_ROR_ 28.4 [27.0, 31.6], pre-ROR 35.7 [31.3, 41.6], *p* < 0.0004) and again from pre- to post-ROR (post-ROR 46.7 [44.0, 49.2], *p* < 0.0001).

### Flattening of Attractors

We plotted 3D point clouds (attractors) constructed from time-delayed embeddings of the 20 s EEG clips at 5 ms delays (shown both at the same scale Figure 1D and independently auto-scaled Figure 1E). Previous analyses of cortical EEG activity have shown flattening of attractors following anesthetic induction with isoflurane at loss of responsiveness in human surgical patients (Watt and Hameroff, 1988) and post loss of righting reflex in micro-EEGs in rats (MacIver and Bland, 2014). More specifically, these 3D attractors appeared to become more ellipsoidal and thinner compared to their spherical, awake counterparts. The opposite trend has been observed during recovery of responsiveness using sevoflurane in humans (Walling and Hicks, 2006). Here, we tested whether similar shape changes are observed following the unique anesthetic exposure combination of N_2_O and remifentanil. Figure 1D shows pre- and post-LOR attractors at the same viewing angle and with the same axes. By viewing attractors using similar axes one can observe changes both in scale (attractors are larger after LOR) and shape (spherical pre-LOR attractors become more ellipsoidal post-LOR). Auto-scaling the attractors as in Figure 1E isolates and emphasizes the shape change. In Figure 6, we show examples of six patient attractors for the same 20 s clips selected for pre- and post-LOR and pre- and post-ROR. Attractors are depicted at the same viewing angle but have been auto-scaled individually to the same maximum x, y and z coordinates to better illustrate their shape change. We observed a similar flattening of the attractors during LOR to verbal stimuli (LOR) in patients (Figure 6). Attractors composed from the pre-LOR 20 s EEG clips in Figure 6 are already flattening as patients were responding to the N_2_O and remi for several minutes. The average time between induction and LOR to verbal stimuli was 4 min (with a standard deviation of 1.4). However, despite the attractors already having been flattened pre-LOR, we still observed significantly more flattening and more ellipsoidal shapes post-LOR (Figure 6). During ROR to verbal stimuli we observed slightly thicker, ellipsoidal attractors compared to post-LOR, which became thicker still and more spherical at post-ROR (Figure 6).

As described above, we quantified this shape change by fitting the 3D attractors with ellipsoids and measuring the ratio between the lengths of the minor and major axes (radius ratio). We observed a significant decrease in the radius ratio between pre- to post-LOR (Figure 4C, *p* < 0.0001) and a further significant decrease in between post-LOR to High-Remi (Figure 4C, *p* = 0.0048). Additionally, we observed a significant increase between High-Remi and pre-ROR (Figure 4C, *p* = 0.0002) as well as a further significant increase from pre- to post-ROR (Figure 4C, *p* = 0.0029). Clearly the attractors distinguished all of the different anesthetic states reliably.

## Discussion

We analyzed effects of the unique combination of N_2_O and remi on the EEG of surgical patients at different clinically relevant time points during their anesthetic exposure. This included time points at loss and ROR transitions and during the deepest level of anesthesia (High-Remi). We characterized the spectral changes that occurred during these periods as well as the discriminability of the spectral edge frequency, BIS values, and our new radius ratio analysis that quantifies the shape changes we observe in point clouds constructed from time-delay embeddings of the EEG signal.

The original purpose of the study on which our retrospective data analysis was conducted was to determine the blood concentration of remifentanil for which there was a 50% probability of adequate anesthesia during abdominal surgery (C_b50_) with 66% N_2_O (Drover and Lemmens, 1998). In the original 1998 publication, Drover and Lemmens reported C_b50_ values of 4.1 ng/ml in men and 7.5 ng/ml in women. Additionally, the C_b50_ values for prostatectomy, nephrectomy, and other abdominal procedures were 3.8, 5.6, and 7.5 ng/ml, respectively.

Previous EEG examinations of N_2_O administration alone have reported significant decreases in total power following LOR and increases in power upon recovery (Foster and Liley, 2011, 2013). Here we report contrary results for both the full spectrum and band-limited total power calculations, likely due to the co-administration of remi with N_2_O. We reason this is largely due to the reduction of high frequency components of the EEG signal in the beta and gamma range, which is typically produced by remi (Hoffman et al., 1993) and contrary to what has been observed with N_2_O administration (Yamamura et al., 1981; Rampil et al., 1998; Foster and Liley, 2011, 2013). This is consistent with our SEF95 estimates from both full spectrum and band-limited ranges. The band-limited (30 Hz cutoff) was calculated and fewer significant distinctions were observed between states indicating the importance of the contribution of bands above 30 Hz. Interestingly, we observe a peak in the percentage of theta activity in the EEG before patients recover their response to verbal commands; this was higher than any percentage of theta during other states. This “withdrawal” or “washout” effect from N_2_O has been reported for 50% administration (Rampil et al., 1998), as well as lower concentrations of 20% and 40% (Foster and Liley, 2011). We observed this change 2.5 (±1.6) min after N_2_O administration is discontinued, and in all patients prior to ROR. Remi was discontinued at different times before and after patients recovered their response to verbal stimuli. In one-third of the patients, remi was discontinued an average of 2.6 (±1.8) min prior to ROR and in the other two-thirds of patients remi was discontinued 3.4 (±2) min after ROR. This means that this significant increase in theta activity prior to recovery does not appear to be affected by the presence of remi even after the patient has recovered their response to verbal commands.

We expected to see a significant decrease in delta and increase in beta power between pre- and post-ROR given the significant fluctuations in the opposite directions that we observed during pre- and post-LOR. We can formulate several hypotheses as to why we did not observe this in our data. The first is the confluence of effects from the agents administered. We observe increases in the percentage of delta activity at LOR that was maintained at High-Remi, similar to what has been described previously about remi influences on EEG activity (Hoffman et al., 1993). The percentage of delta significantly decreases between High-Remi and pre-ROR indicating that the lightening of the remi concentration is decreasing the delta in the EEG. Thus, there is likely less of a difference in blood concentration levels of remi pre- and post-ROR compared to pre- and post-LOR and thus less influence on the EEG activity. We also reported that remi was discontinued at different times for different patients, which may have influenced these results. Lastly, our test for the ROR to verbal stimuli may have occurred after a patient had exhibited other signs of arousal including heart rate and blood pressure increases, movement, lacrimation, or diaphoresis. More awake-like activity can be clearly seen in the pre-ROR period of our spectrograms indicating that patients are lightening prior to being able to respond to verbal stimuli. Indeed, patients can have other cognitive functions recover prior to being able to respond to verbal commands (Williams and Sleigh, 1999).

Midazolam (1- to 2-mg intravenous dose) was given an average of 26 (±13) min prior to the onset of the anesthesia. The influence of midazolam on the EEG activity (namely increases in beta power) at the dose administered can last ~1–2 h after administration; however, the increase in beta power caused by midazolam rapidly declines after administration (Veselis et al., 1993; Greenblatt et al., 2004). It is possible that the decrease in beta activity we report during LOR could be partially attributed to declining effect site concentrations of midazolam. EEG effects in the beta band range during our LOR time point of the midazolam decrease and remi increase are indistinguishable. Additionally, co-administration of midazolam and remi decrease BIS values as a patient transitions from an awake state to an unconscious state (Haenggi et al., 2009), so the BIS effects would be indistinguishable at this time point as well. In our case, the effects of these two agents during LOR would be complementary as midazolam is decreasing and remi is increasing.

Previous attempts to use the BIS to determine depth of anesthesia, using N_2_O, reported that the BIS number does not change with LOR due to the maintenance of high frequency content in the EEG post anesthetic induction (Yamamura et al., 1981; Rampil et al., 1998; Barr et al., 1999; Hirota et al., 1999; Foster and Liley, 2011, 2013). In contrast, our results show that the BIS number is significantly reduced and able to separate out loss and ROR in patients when they are given both N_2_O and remifentanil together. This is likely due to the effects of remifentanil increasing the lower frequency power and, more importantly, decreasing the power in higher frequency bands (Lozito et al., 1994; Ruskin et al., 2013). Much as has been reported in propofol studies where remi was used as an adjuvant agent (Koitabashi et al., 2002), we observe that remi also decreases BIS values during response transitions, which are not observed during N_2_O administration alone (Rampil et al., 1998). Addition of remifentanil to N_2_O administration may also improve the ability of other monitoring algorithms, such as entropy, to detect LOR and ROR in surgical patients (Anderson and Jakobsson, 2004; Sleigh and Barnard, 2004; Anderson et al., 2005).

One issue with our data, and indeed of any EEG measurement, is muscle contamination. Visually inspected clips were manually selected to rule out any contamination from large muscle artifacts such as eye blinks, teeth-chattering, and eye rolls. However, muscle activity is a pernicious and precarious issue in EEG as electrical activity generated from facial and neck muscles can extend from the 20 Hz to 200 Hz frequency ranges (Shackman et al., 2009; Claus et al., 2012; Muthukumaraswamy, 2013). Consequently, it is important not to cut out higher frequencies as they correlate with cognition and are important components of the signal for judging anesthetic depth (Sleigh et al., 2001; Muthukumaraswamy, 2013). In our data set as well as others, high frequency activity is an important marker of consciousness. We calculated an output measure from the A-1000 known as EMGlow which per the manufacturers manual is the absolute power in the 70–100 Hz range. This number is calculated from the same electrodes as the EEG activity and could also be considered high gamma activity which is correlated with cognition in the absence of EMG activity (Hudetz et al., 2011; Lachaux et al., 2012). We found that this number EMGlow, like the BIS, decreased with increasing anesthetic depth and then increased when patients recovered from anesthesia. This is precisely the trend one would expect for both muscle contamination and high gamma activity with our combination of anesthesia administration. Note this is contrary to previous results reporting sole N_2_O administration increasing EMGlow (Rampil et al., 1998) and again likely reflects remi predominantly modulating the low frequency EEG activity.

Previous work has demonstrated that 3D time-delayed embeddings of the EEG signal transition from spherical balls to flatter shapes as patients lose consciousness during surgical procedures, and return to spherical configurations upon recovery (Watt and Hameroff, 1988; Walling and Hicks, 2006; MacIver and Bland, 2014). Shape changes have been reported using sevoflurane (Walling and Hicks, 2006) and isoflurane co-administered with fentanyl (Watt and Hameroff, 1988) in humans and with isoflurane in rats (MacIver and Bland, 2014). We observed the same flattening and re-inflation of attractors using the combination of N_2_O and remi in human surgical patients. For this study, we wanted to determine whether shape changes could be observed with time-delayed embeddings. The nuances of our frequency measures indicate that it would be useful for future work to test the contribution of different frequency bands to the embedding structure. We are confident that the results are not due to muscle artifact as similar changes have been observed in intracranial recordings from rats (MacIver and Bland, 2014). We developed a novel analysis measure to characterize this observation, fitting the 3D attractor with an ellipsoid and calculating the ratio of the lengths of minimum and maximum symmetry axes. This new analysis shows significant differences between clinically relevant behavioral transitions during surgical procedures. Our results suggest that the observed shape change of time-delayed embeddings may be a definitive measure of anesthetic depth; however, more testing in diverse patient populations with diverse anesthetic protocols is needed to validate this claim.

## Conclusion

We have shown that addition of remifentanil to N_2_O anesthesia improves performance of spectral and BIS values to distinguish anesthetic states when compared to previous work. We also observed similar shape changes in three-dimensional embeddings of EEG activity; whereby, these embeddings change from spheres during awake states to elongated ellipsoids during anesthetized states. We introduced a novel characterization of these shape changes, the radius ratio, which significantly distinguished between anesthetic states better than most spectral measures and as well as the BIS.

## Author Contributions

MBM, DRD and SLE designed the work. DRD acquired the data. CMD organized the anesthesia records for analysis. SLE, MBM and NTO analyzed the data. SLE wrote the manuscript. All authors contributed to the intellectual content with rounds of review. All authors approved the final version of the manuscript for publication.

## Conflict of Interest Statement

DRD is a consultant for Masimo Inc. The other authors declare that the research was conducted in the absence of any commercial or financial relationships that could be construed as a potential conflict of interest. The reviewer SH and handling Editor declared their shared affiliation.

## Footnotes

^1^http://opentci.org/code/stanpump

^2^http://chronux.org/
